# Approaches to Improve Endometrial Receptivity in Case of Repeated Implantation Failures

**DOI:** 10.3389/fcell.2021.613277

**Published:** 2021-03-16

**Authors:** Antonis Makrigiannakis, Fanourios Makrygiannakis, Thomas Vrekoussis

**Affiliations:** Department of Obstetrics and Gynecology, Medical School, University of Crete, Heraklion, Greece

**Keywords:** repeated implantation failures, HCG, PBMC, PRP, microbiome, G-CSF, atosiban, growth hormone

## Abstract

Repeated implantation failures are a constant challenge in reproductive medicine with a significant impact both on health providers and on infertile couples. Several approaches have been proposed so far as effective; however, accumulative data have clarified that most of the treatment options do not have the evidence base for a generalized application to be suggested by the relevant societies. Implantation failures are attributed to either poor quality embryos or to defected endometrial receptivity. The current review aims to summarize in a systematic way all the new trends in managing RIF via interference with endometrial receptivity. The authors focus mainly, but not exclusively, on endometrial injury prior to embryo transfer and endometrial priming with autologous cells or biological agents. To this direction, a systematic search of the Pubmed database has been conducted taking into account the emerged evidence of the last two decades. All the suggested interventions are herein presented and analyzed in terms of reproductive outcomes. It is evident that properly powered and designed randomized trials are needed to support a new standard approach in RIF treatment that will safely be incorporated in national and international guidelines.

## Introduction

Repeated implantation failure (RIF) is one of the main challenges in human reproduction. Due to the fact that RIF was initially considered a rather heterogeneous entity, a definition was difficult to establish. It is however accepted that RIF is defined as “the failure to achieve a clinical pregnancy after transfer of at least four good-quality embryos in a minimum of three fresh or frozen cycles in a woman under the age of 40 years” (Coughlan et al., 2014a). This definition is further challenged upon the number and the type of embryos transferred (number of cleavage embryos vs. number of blastocysts), along with the definition of the primary endpoint for a cycle to be successful (biochemical vs. clinical pregnancy) (Cakiroglu and Tiras, 2020). Even so, the even existence of RIF as a clinical entity is under doubt (Ben Rafael, 2020). Due to the diversity of the RIF definitions, data on RIF incidence is rather restricted (Bashiri et al., 2018).

RIF is a burden both for the health providers and the couples. Health providers are required to proceed to assisted reproduction techniques with rather small success rates, while the couples are overloaded with psychological stress (Coughlan et al., 2014b; Stanhiser and Steiner, 2018), not to mention the financial pressure due to the repeated cycles. It is thus imperative for health providers to employ novel tools aiming to improve the reproductive outcome. So far, only hysteroscopy to treat endometrial pathology (Mao et al., 2019), and treatment of hydrosalpinges (Coughlan, 2018) have been proven significantly beneficial and as such they have been incorporated in standard care. However, several approaches have emerged in the literature claiming to act like the “Holy Grail” in management of unexplained RIF.

Herein, we present a systematic effort to present the existing evidence on most of the novel approaches aiming to improve implantation and thus reproductive outcomes in case of unexplained RIF.

## The Endometrial Pathophysiology of RIF

The etiology of RIF can be attributed to dysfunction of the two major players of implantation, namely the embryo and the endometrium. As far as the embryo is concerned, poor quality embryos or sperm along with parental chromosomal anomalies are the main causes of an embryo failing to implant (Coughlan et al., 2014a). Such issues of poor quality gametes can be easily diagnosed prior to IVF. On the other hand, deranged endometrial receptivity is more difficult to evaluate; apart from hysteroscopy to assess the endometrial cavity, only very recently molecular diagnostic arrays have been available in order to predict an IVF candidate as a RIF patient; however, the evidence is rather weak for such approach to be established in clinical practice (Bassil et al., 2018). The molecular signature of RIF is constantly under investigation; a recent report has shown that a molecular signature of 303 genes extracted from endometrial sampling could safely discriminate between normal and RIF individuals (Koot et al., 2016). Such approaches, although promising, need further validation in order to be released for clinical practice.

### The Endometrial Pathophysiology of RIF: Well-Established RIF Causes

#### Anatomical Disorders

RIF may be attributed to anatomical disorders that distort the endometrial cavity, being undiagnosed before IVF treatment. In that context, fibroids have been reported as negative prognosticators to IVF success (Wang et al., 2018; Rikhraj et al., 2020), altering endometrial receptivity by modifying HOXA10 and LIF expression (Makker et al., 2017; Kara et al., 2019; Pier et al., 2020). Endometrial adhesions as a result of infection or prior surgical procedure may also be considered to be associated with thin endometrium and low receptivity potential (Wang et al., 2020). Finally, hydrosalpinges are well-accepted as a cause for RIF, since the inflammatory fluid may affect both the embryo and the endometrium (Volodarsky-Perel et al., 2019). Of note is the fact that patients with the above mentioned anatomical disorders may receive surgical treatment prior to IVF with significantly improved reproductive outcomes.

### Unexplained RIF: Proposed Endometrial Pathophysiology

#### Immunological Disorders

The immunological profile of the receptive endometrium presents several characteristics that seem to be impaired in case of implantation failure. The first cellular population to be considered of interest was the natural killer (NK) cells, due to their ability to destroy allogenic cellular signals. It has been previously reported that increased numbers and activity of peripheral NK cells are associated with a negative pregnancy outcome (biochemical pregnancy or miscarriage) (Yamada et al., 2003). To the same direction, a parallel increase in peripheral and uterine NK cell numbers and NK activity was found in women diagnosed as RIF (Santillán et al., 2015). However, the role of the NK cells in human reproduction is quite complex; NK cells constitute a rather diverse cellular population making the discrimination between peripheral and uterine NK cells rather difficult. Interestingly, it was shown that even uterine NK cells may be divided into three subsets with different immunological properties (Vento-Tormo et al., 2018). Perhaps, due to this diversity, the first meta-analysis in the field, including studies assessing NK cell biology prior IVF treatment, showed no significant difference in NK cell count and activity between fertile and infertile women (Seshadri and Sunkara, 2014). Emerging evidence now put in doubt the initial notion that NK cell density and activity may predict RIF (Donoghue et al., 2019; Zhang et al., 2020), while a recent meta-analysis highlights that studies reporting interventions based on NK status are heterogeneous and lack the quality to produce solid evidence (Woon et al., 2020).

The role of differential expression of several cytokines in implantation has been well described in the literature. A constant shift to a Th1 cytokine pro-inflammatory profile contributes to implantation failure or miscarriage (Liang et al., 2015). On the contrary, a shift toward a Th2 anti-inflammatory cytokine profile supports implantation and early fetal development. Interestingly, a successful implantation requires a T-regulatory (Treg) cell profile, while a shift toward a Th17 phenotype is associated with poor reproductive outcomes (Ali et al., 2018). It is reported that up to 80% of RIF cases may present with an abnormal cytokine profile (Lédée et al., 2016). It must be pointed out though, that, even well-studied, the above mentioned findings should be met with caution. The correlation of the immune profile with reproductive success has not been principally validated; it has been proven within special research settings.

#### Non-immunological Disorders

Several signaling pathways have been reported as impaired in case of repeated implantation failures. A recent transcriptome analysis has revealed that, in case of RIF, leukemia inhibitory factor (LIF) was reduced along with the expression of S100 calcium binding protein P (S100P), Chemokine (C-X-C motif) ligand 13 (CXCL13), SIX homeobox 1 (SIX1) and signal transducer, and activator of transcription 3 (STAT3) (Choi et al., 2016). Additionally, the endometrium of RIF patients has been characterized as of low MUC1 expression, this being an independent prognosticator of implantation failure (Wu et al., 2018). Furthermore, platelet and endothelial cell adhesion molecule 1 (PECAM1) and transforming growth factor β1 (TGF-β1) were also significantly reduced in RIF (Guo et al., 2018). Apart from altered implantation markers, it has been previously shown that prostaglandins’ synthesis is deranged in case of RIF, implying a defective endometrial inflammation in favor of implantation failure (Achache et al., 2010; Demiral Keleş et al., 2020). Finally studies evaluating metabolomics (RoyChoudhury et al., 2016) and microRNAs (Shi et al., 2017) have shown that RIF may be featured by a significant different profile that could be associated with poor reproductive outcome.

#### Chronic Endometritis

Chronic endometritis (CE) is an emerging entity considered to negative affect reproductive outcomes in case of IVF treatment. Especially in RIF, CE has been reported at an incidence of ranging from 14 to 30% with decreased pregnancy success rates (Quaas and Dokras, 2008; Bouet et al., 2016). EC diagnosis is rather complicated. Endometrial cavity assessment is initially performed via hysteroscopy, recognizing subtle endometrial lesions like micropolyps, stromal edema and profound vascularity attributed to inflammatory angiogenesis (Gkrozou et al., 2020). The gold standard in establishing CE diagnosis is the recognition of increased plasma cell density in the endometrial stroma, either by standard histology (Kasius et al., 2012), or even better, by immunohistochemically marking plasma cells with anti-CD163 (Fan et al., 2019; Li et al., 2020; Xu et al., 2020). A complementary approach is endometrial sampling for microbial culture. The most common pathogens identified are so far *group B Streptococcus, Escherichia Coli, Streptococcus Faecalis, Mycoplasma* (Cicinelli et al., 2015). Of note is the constant risk of sample contamination with vaginal or cervical pathogens. CE is considered to affect endometrial receptivity via establishing a dysbiotic endometrial environment featured by dense lymphocyte populations along with a shift toward inflammatory cytokine profiles (Th1/Th17) (Mor and Kwon, 2015; Al-Nasiry et al., 2020).

#### Thin Endometrium

Although there is no universal consensus about the threshold of thin endometrium, an endometrial thickness below 8 mm is generally accepted as characteristic for thin endometrium. Thin endometrium is a risk factor for implantation failure (Liu et al., 2018). Thin endometrium could be initially attributed to previous endometrial infection or intra-cavitary intervention. In the absence of an obvious cause, it is suggested that it could be the end result of defective angiogenesis, depriving the endometrium from the necessary nutrients and oxygen (Miwa et al., 2009). Several approaches have been proposed including adhesiolysis and estrogen treatment (Lebovitz and Orvieto, 2014). However, thin endometrium remains a challenge and as such novel approaches need to be devised and properly evaluated.

#### Dysbiotic Microbiome: A Novel Pathophysiologic Approach in RIF

During the last decade, with the technological evolution of new generation sequencing, it became feasible to evaluate the microbiome of the reproductive system. It is currently known that the vagina microbiome is considered of low diversity, having predominantly lactobacillus species (Ravel et al., 2011). Lactobacillus is the natural guardian of the vagina, since it metabolizes glycogen released by vaginal epithelium to lactic acid, securing a low pH which in turn inhibits the growth of local pathogens. Several controlled studies have been published, demonstrating that altered vaginal or even endometrial microbiome could be associated with poor reproductive outcomes. Of note is the study by Moreno et al. (2016) showing that pathological endometrial microbiota are associated with implantation failure. These studies have been recently systematically reviewed with the conclusion being supportive for the altered lactobacillus population to be a potential cause of impaired endometrial receptivity (Bracewell-Milnes et al., 2018). Normal endometrial microbiome is expected to be of low biomass, exerting a moderate local immune stimulation in favor of normal tissue remodeling (Einenkel et al., 2019). Furthermore, it is expected to support the endometrium via production of metabolites, while concurrently it blocks pathogen migration via spatial antagonization (Benner et al., 2018). On the contrary, dysbiotic microbiota are featured by abundancy and a powerful immune stimulation with a local destructive result (Einenkel et al., 2019). It has been noted that normally, the endometrial microbiome contributes to a cytokine profile toward Th2/Treg immunity (Al-Nasiry et al., 2020). On the other hand, dysbiosis, induces a Th1/Th17 profile exerting a negative effect on tissue remodeling and trophoblast invasion (Al-Nasiry et al., 2020). Finally, the dysbiotic microbiome contributes to local oxidative stress with detrimental effect on endometrial cell homeostasis (Baker et al., 2018).

Despite the initiated enthusiasm, there are several issues regarding the procedure of sampling and evaluating endometrial microbiota. It has been reported that endometrial microbiome may fluctuate according to the circulating estrogen and progesterone (Molina et al., 2020). Additionally, it may be altered by infectious agents, increasing age, physical activity, pregnancy and childbirth (Molina et al., 2020). More importantly, endometrial microbiome evaluation is under the influence of technical details that need standardization. There is always the risk of contamination by the vaginal microbiome (Salter et al., 2014; Glassing et al., 2016). This demands a careful sampling along with setting proper negative controls (Kim et al., 2017). The platform used for sequencing may also affect the results (Clooney et al., 2016). Moreover, since endometrial microbiome is of low biomass, special DNA isolation kits are needed in order to minimize the risk of misinterpretation of the results along with the risk of inserting bias via the statistical method applied (Eisenhofer et al., 2019; Weyrich et al., 2019). Finally, the results need further critical analysis, since detecting 16s rRNA does not mean that the strains identified are necessary viable or abundant. To this direction, no “core endometrial microbiome” has been presented so far, nor has this been correlated with normal/fertile endometrium or any uterine pathology (like polyps or retarded decidualization).

## Materials and Methods

The aim of the current study was to highlight novel approaches in the field of unexplained RIF treatment. The Pubmed database was screened with the following searches: (“Endometrial injury” OR “Endometrial scratching”), [(“HCG” OR “human chorionic gonadotropin”) AND (“intrauterine” OR “infusion” OR “injection” OR “administration”)], [(“PBMC” OR “peripheral blood mononuclear cells” OR “peripheral blood monocytes”) AND (“intrauterine” OR “infusion” OR “injection” OR “administration”)], [(“G-CSF” OR “Granulocyte colony stimulating factor”) AND (“IVF” OR “assisted reproduction”)], [(“atosiban” AND (“IVF” OR “assisted reproduction”)], [(“GH” OR “Growth hormone”) AND (“IVF” OR “assisted reproduction”)], [(“PRP” OR “platelet-rich plasma”) AND (“IVF” OR “assisted reproduction”)], (“antibiotics” AND “chronic endometritis”), [(“microbiome” AND (“IVF” OR “assisted reproduction”)]. The publications were screened by title relevance and thereafter by abstract relevance. Only controlled trials and meta-analyses were included. Meta-analyses’ references were also screened for Pubmed publications.

## Approaches to Improve Endometrial Receptivity in Unexplained RIF Patients

### The Role of Endometrial Injury in Improving Reproductive Outcomes in Women With RIF

The concept of performing an endometrial injury as a means of improving endometrial receptivity has been reported by Barash et al. (2003). The authors demonstrated, for the first time, a significant improvement both in implantation and clinical pregnancy rates. This report triggered a massive positive reaction by clinical research teams aiming to incorporate this simple and low cost approach in their everyday clinical practice. As a result, both basic science teams and clinicians published a significant number of studies in the view both to delineate possible potential pathophysiological mechanisms, along with producing solid clinical evidence for endometrial injury to be accepted as a therapeutic procedure.

As far as basic science is concerned, endometrial injury has initially been proposed to induce an aseptic inflammatory reaction possibly shifting the endometrial immune profile toward a Th2/M2 state (Granot et al., 2012). It was, thus, shown that endometrial injury may up-regulate the endometrial expression of several pro-decidualization molecules, including MUC-1, crystalline aB, APOD, and PLA2 (Kalma et al., 2009). MUC-1 is known to be up-regulated by progesterone affecting endometrial receptivity, acting at the same time as an independent receptivity marker in case of RIF (Wu et al., 2018). To the same direction, it has been shown that endometrial injury may induce uroplakin Ib expression, a molecule up-regulated mainly during the window of implantation (Kalma et al., 2009). Further studies have shown an induction of the endometrial repair mechanism involving the up-regulation of TNFα (Gnainsky et al., 2010). This in turn initiates the chemo-attraction of monocytes and dendritic cells, thus increasing the number of endometrial macrophages in favor of the implantation process, since they trigger the endometrial expression of osteopontin, a well-known receptivity marker (Gnainsky et al., 2015). More recent studies have also highlighted the activation of local angiogenesis as this is identified by elevated expression of VEGF, a phenomenon attributed to elevated HIF-1α expression as a result of inflammatory hypoxia (Yu et al., 2019). The complex network of aseptic inflammation and angiogenesis mediators has been considered as a positive contributor to receptivity (Yang et al., 2019).

In the field of clinical trials, many non-randomized and randomized controlled trials have been published (summarized in Table 1). All the trials have employed endometrial injury following various protocols in terms of: (a) number of procedures prior to embryo transfer, and (b) the timing of the procedure (follicular or luteal phase or both). Following the time line of the trials published since 2003 up to present, it can be recognized that initially the results were very supportive of the procedure and this was further presented in the first meta-analyses presented in 2012 (El-Toukhy et al., 2012; Nastri et al., 2012; Potdar et al., 2012). The initial enthusiasm was followed by comments regarding the quality of the included randomized trials, along with concerns upon a possible selection bias (Simón and Bellver, 2014). Since then, further studies of different sizes and methodology have been added, increasing the heterogeneity. Due to the lack of uniformity in performing the procedure, the most recent meta-analyses have noted the weaknesses of the randomized trials, pooling data that lead to rather discouragement (Gui et al., 2019; Sar-Shalom Nahshon et al., 2019; van Hoogenhuijze et al., 2019; Vitagliano et al., 2019). To this direction, a critical review of the randomized controlled trials published so far, revealed several issues in trials’ design, underlying that caution is needed especially when pooling low-quality evidence (Li et al., 2019). Very recently, a properly powered randomized trial has been published (Lensen et al., 2019a). Having recruited 1,364 patients randomized to receive or not an endometrial injury prior to embryo transfer, the authors state that performing an endometrial injury in everyday practice does not significantly alter the reproductive outcomes (Lensen et al., 2019a).

**TABLE 1:** Salient features of the included studies on endometrial injury as an intervention in improving endometrial receptivity.

Year	PMID	Publication type	Participants	RIF	Outcome
2020	32468267	RCT	352	Not exclusively	Non-significant
2020	32372078	RCT	200	Not exclusively	Negative–premature end
2020	32216503	Non-randomized	518	Not exclusively	Significant
2020	32003122	RCT	200	YES	Significant
2020	31897673	Non-randomized	300	Not exclusively	Significant in RIF
2019	31843072	RCT	304	Not exclusively	Significant in RIF
2019	31532321	Non-randomized	62	YES	Significant in RIF
2019	31450870	Non-randomized	137	Not exclusively	Non-significant
2019	31405721	RCT	239	YES	Significant in RIF
2019	30895265	Meta-analysis	2537	Not exclusively	Non-significant
2019	30683590	Meta-analysis	1354	Not exclusively	Non-significant
2019	30673547	RCT	1364	Not exclusively	Non-significant
2019	30661093	RCT	51	Not exclusively	Non-significant–premature end
2019	30515920	Non-randomized	266	Not exclusively	Significant
2019	30421580	Meta-analysis	4057	Not exclusively	Non-Significant in RCTs Significant overall
2019	30388238	Meta-analysis	1260	Not exclusively	Non-significant
2019	30496529	RCT	191	Not exclusively	Non-significant–Premature end
2018	29048754	RCT	300	Not exclusively	Significant
2018	30196966	Meta-analysis	1468	YES	Significant in RIF
2017	29259469	RCT	77	YES	Significant in RIF
2017	28964963	RCT	80	Not exclusively	Non-significant
2017	28551840	RCT	144	Not exclusively	Significant
2017	28511086	RCT	111	Not exclusively	Non-significant
2017	28447502	Non-randomized	576	Not exclusively	Non-significant
2017	28397981	RCT	106	Not exclusively	Significant
2017	28386815	Non-randomized	429	YES	Significant in RIF
2017	28612975	RCT	169	Not exclusively	Non-significant
2016	28101111	RCT	120	YES	Non-Significant in RIF
2016	27910711	Non-randomized	103	YES	Significant in RIF
2016	27363928	RCT	120	Not exclusively	Non-significant
2016	27738660	RCT	63	Not exclusively	Negative
2016	27525329	RCT	93	Not exclusively	Non-significant
2016	27296541	Meta-analysis	1512	Not exclusively	Uncertainty due to low quality
2016	27294218	RCT	400	Not exclusively	Significant
2016	27258405	Non-randomized	345	YES	Significant in RIF
2016	27146582	RCT	360	Not exclusively	Significant
2016	26342054	RCT	154	Not exclusively	Significant
2015	26752857	RCT	60	YES	Significant implantation rate
2015	26538858	RCT	251	Not exclusively	Significant
2015	25803542	Meta-analysis	2128	Not exclusively	Significant
2015	25561347	RCT	387	Not exclusively	Significant only in RIF
2015	26344351	RCT	332	Not exclusively	Non-significant
2014	25469138	RCT	144	Not exclusively	Non-significant
2014	25205759	RCT	300	Not exclusively	Non-significant
2014	25064410	Non-randomized	737	Not exclusively	Non-significant in RIF
2014	24791967	Non-Randomized	80	Not exclusively	Non-significant
2014	24289893	Non-randomized	118	Not exclusively	Significant
2013	24639710	RCT	217	Not exclusively	Significant
2013	23754314	RCT	158	Not exclusively	Significant
2013	23106834	RCT	101	Not exclusively	Significant
2013	23494199	RCT	150	Not exclusively	Significant
2013	24283157	Non-randomized	89	YES	Significant in RIF
2012	25246928	Non-randomized	83	Not exclusively	Significant
2012	23063812	Meta-analysis	2062	YES	Significant in RIF
2012	22885017	Meta-analysis	901	Not exclusively	Significant
2012	22943664	RCT	36	YES	Negative in RIF
2012	22835632	RCT	200	YES	Significant in RIF
2011	22014336	Non-randomized	30	Not exclusively	Significant
2011	26396577	Non-Randomized	74	Not exclusively	Non-Significant
2010	20607003	RCT	100	Not exclusively	Significant
2010	19568761	RCT	77	Not exclusively	Negative
2009	20070722	RCT	115	YES	Significant in RIF
2008	17681303	RCT	121	Not exclusively	Significant
2007	17197286	Non-randomized	117	YES	Significant in RIF
2003	12798877	Non-randomized	134	Not exclusively	Significant

The evidence produced from this study (Lensen et al., 2019a), has initiated a long series of debates in terms of the endometrial injury application, along with the ethical dilemma of offering a procedure proven as useless or even possibly harmful (Yeung et al., 2014; Frantz et al., 2019; Lensen et al., 2019b; Mackens et al., 2020). This is especially important in case of selected groups receiving assisted reproduction treatments like women with RIF. Although Lensen et al. reported that endometrial injury was not efficient in women with RIF (Lensen et al., 2019a), this result was extracted by a sub-group analysis of the population. Despite the fact that the study was properly powered to identify a significant difference of 15% between the study and the control groups, there are always methodological issues in sub-group analyses, mainly due to lack of stratified randomization (VanderWeele and Knol, 2011; Lensen et al., 2019b). The clinical evidence to support endometrial injury in women with RIF is based on significantly fewer studies compared to the total number of studies published so far (see Table 1). The heterogeneity of these studies was addressed in a previous systematic review (Panagiotopoulou et al., 2015). Most of the studies have been summarized in a recent meta-analysis which clearly demonstrates that in case of women with RIF, endometrial injury may significantly improve reproductive outcomes (Vitagliano et al., 2018a). Interestingly, the same research group has demonstrated in a separate meta-analysis that the positive effect of the procedure does not exist in case of women receiving their first IVF treatment (Vitagliano et al., 2019). This is in line with the report of Lensen et al. (2019a), strengthening the notion that endometrial injury should not be an everyday practice anymore. A properly designed randomized controlled trial is expected to delineate whether offering endometrial injury is beneficial to women with RIF. Until then, the patients should be properly informed about the potential benefits of the procedure and the lack of solid evidence.

### Intrauterine Administration of Human Chorionic Gonadotropin (HCG)

The concept of administering HCG in the uterine cavity before embryo transfer was based on evidence produced during the last two decades. It is well-established that HCG is the first molecule to participate in the cross-talk between the embryo and the maternal decidua. HCG is expressed even at the stage of the 8-cell embryos (Bonduelle et al., 1988; Lopata and Hay, 1989), following a specific pattern of augmentation during implantation and trophoblast invasion, inducing the differentiation of the cytotrophoblast to syncytiotrophoblast. At the same time a switch from the standard HCG to the hyperglycosylated HCG isoform (H-HCG) is characteristic during blastocyst hatching, with the latter being involved in extravillous trophoblast proliferation and invasion (Cole, 2010; Guibourdenche et al., 2010). It is thus evident that the concurrent actions of HCG and HCG-H enhance placentation and thus early fetal development.

The role of HCG was further established in reproductive physiology by several reports investigating the impact of HCG on the endometrium during decidualization, implantation and trophoblast invasion. HCG was found to induce α-smooth muscle actin in endometrial stroma fibroblasts (Fazleabas et al., 1999), a fact linked to decidualization. Additionally, HCG was reported to be a regulator of glycodelin (Toth et al., 2008) and progesterone receptors’ expression (Tapia-Pizarro et al., 2017), both regulating decidualization. To the same direction HCG was demonstrated as a facilitator of implantation, since several receptivity-related molecules like VEGF, HOXA-10, and galectin-3 are up-regulated by HCG (Fogle et al., 2010; Yang et al., 2013). Interestingly, HCG was recently reported to mediate chemo-attraction (Schumacher et al., 2009) and differentiation of T-regulatory (Treg) cells (Diao et al., 2017), while inducing T-cell apoptosis via the Fas/FasL system, thus acting as a local immuno-modulator during implantation and trophoblast invasion (Kayisli et al., 2003). Concurrently, HCG was shown to promote trophoblast invasion by upregulating the pro-invasive VEGF, LIF and MMP-9 (Licht et al., 1998; Fluhr et al.,2008a,b), while down-regulating tissue inhibitors of metalloproteinases (Fluhr et al., 2008a; Tapia-Pizarro et al., 2013). Interestingly, HCG may also pose a negative effect on the endometrium; a prolonged low-dose HCG administration was proved to downregulate the LH-HCG receptor, possibly making the endometrium unresponsive to blastocyst secreted HCG (Evans and Salamonsen, 2013).

All the above established an evidence base for clinical trials. A moderate number of clinical trials have been performed so far (summarized in Table 2), with the majority of them being randomized controlled trials (Mansour et al., 2011; Hong et al., 2014; Santibañez et al., 2014; Zarei et al., 2014; Aaleyasin et al., 2015; Wirleitner et al., 2015; Dehghani Firouzabadi et al., 2016; Navali et al., 2016; Huang et al., 2017a; Mostajeran et al., 2017; Boonsuk et al., 2018; Hafezi et al., 2018; Laokirkkiat and Thanaboonyawat, 2019). Despite the anticipation for solid evidence, an in-depth evaluation reveals high heterogeneity due to different methodologies applied. Indeed, treatment protocols differ in terms of HCG dosage, timing of intrauterine administration and the stage of the embryos transferred. Apart from protocol heterogeneity, patient characteristics differ as well, ranging from infertile women to patients experiencing repeated implantation failures. These differences can initially explain the contradicting results of the published trials along with the opposing conclusions drawn by the meta-analyses performed during the last 5 years. So far four meta-analyses (Ye et al., 2015; Osman et al., 2016; Hou et al., 2018; Gao et al., 2019) and one Cochrane review (Craciunas et al., 2018) have been performed with opposing results. Only 2 out of the 4 meta-analyses present a significant benefit (Ye et al., 2015; Gao et al., 2019). An in-depth analysis of the most recent meta-analyses has revealed differences in the included studies, mainly being reports published as abstracts in proceedings’ volumes possibly published with less stringent criteria in terms of a peer review process. To date, the recent Cochrane review with 4,751 participants is the largest pooled population (Craciunas et al., 2018). The authors consider that a meta-analysis to address the efficacy of intra-uterine HCG administration, is not feasible due to the heterogeneity mostly attributed to the HCG dosage and the stage of the embryo transferred (Craciunas et al., 2018). To the direction of increased heterogeneity, the source of HCG administered (recombinant vs. urinary) could also be pointed out. By performing sub-group meta-analysis, Craciunas et al. (2018) conclude that HCG may improve reproductive outcomes only in case of cleavage embryos after having primed the endometrial cavity with at least 500IU of HCG. Surprisingly, being in line with the findings of Evans and Salamonsen urging for a potential negative effect of HCG in implantation (Evans and Salamonsen, 2013), a recent non-randomized trial reported a significantly negative outcome when HCG was administered in non-RIF patients followed by a fresh embryo transfer (Volovsky et al., 2018).

**TABLE 2:** Salient features of the included studies on HCG as an intervention in improving endometrial receptivity.

Year	PMID	Publication type	Participants	RIF	Outcome
2019	31704529	Meta-analysis	1,432	YES	Significantly Favorable in RIF
2019	31277770	Meta-analysis	2,763	Not exclusively	Significantly Favorable
2019	30659362	Non-randomized	305	YES	Significantly favorable in RIF
2019	30449012	RCT	200	Not exclusively	Significantly favorable only for implantation rates
2018	30291482	Meta-analysis	2,759	Not exclusively	Non-significant
2018	30341915	Meta-analysis	4,751	Not exclusively	Significantly favorable only in case of cleavage embryos and HCG ≥ 500IU
2018	29626233	RCT	180	Not exclusively	Non-significant
2018	29288552	Non-randomized	225	YES	Significantly favorable for RIF
2018	28948440	Non-randomized	1,207	Not exclusively	Significantly unfavorable in FET and women without RIF
2017	28400828	RCT	100	Not exclusively	Non-significant
2016	27921090	RCT	159	Not exclusively	Non-significant
2016	27680029	RCT	158	Not exclusively	Significantly favorable
2017	27449969	RCT	161	YES	Significantly favorable compared to controls. Placebo also improved the outcomes
2016	27317131	Meta-analysis	3,087	Not exclusively	Non-significant
2015	26359294	Meta-analysis	1,387	Not exclusively	Significantly favorable
2015	26141379	RCT	1,186	Not exclusively	Non-significant
2015	25531413	RCT	483	Not exclusively	Significantly favorable
2014	24799855	RCT	182	Not exclusively	Significantly favorable
2014	25234040	RCT	300	Not exclusively	Non-significant
2014	24476536	RCT	210	Not exclusively	Significantly favorable
2011	22047664	RCT	260	Not exclusively	Significantly favorable

The issue of HCG administration in case of women with RIF is even more perplexed. A recent proteomic analysis of women with RIF showed a different proteomic profile compared to fertile controls even in molecules not included in commercially available receptivity assays (Bielfeld et al., 2019). Interestingly it was shown *in vitro* that HCG could alter the proteomic profile in terms of endocytosis, HIF signaling and chemokine production (Bielfeld et al., 2019). This proves that RIF patients constitute a distinct population not to be treated simply as “infertile.” Such an approach dictates clinical trials to be designed exclusively for RIF patients. So far, only one full-paper RCT has been published addressing the issue of HCG efficacy in women with RIF, reporting HCG as significantly beneficial compared to controls; however benefit was shown even from placebo, implying an underlying endometrial injury effect (Huang et al., 2017a). The major core of evidence stems from non-randomized trials. A recent meta-analysis has summarized this evidence, supporting the use of HCG as an endometrial primer prior to embryo transfer in women with RIF (Xie et al., 2019). However, this meta-analysis has included both RCTs (including 2 RCTs published as abstracts) and non-randomized trials, a fact that poses a question upon the level of evidence produced. Further properly powered studies are needed to clarify the role of intrauterine HCG administration as a treatment option in women with RIF. Until such solid evidence emerges, the most prudent approach is to adhere to the findings of Craciunas et al. (2018). Even this is the best existing evidence so far, until properly designed randomized controlled trials verify such finding, HCG is not to be incorporated in clinical practice. It could be offered as a treatment option within the frame of a research protocol.

### Intra-Uterine Administration of Peripheral Blood Mononuclear Cells (PBMC)

Early reports based on *in vitro* and *in vivo* experiments have suggested that PBMC may modulate endometrial receptivity by (a) inducing a Th2 cytokine profile (Hashii et al., 1998), and (b) regulating trophoblast invasion (Nakayama et al., 2002). Further postulations have been expressed: being a heterogeneous cell population (B- and T-lymphocytes, monocytes and macrophages) PBMC were considered ideal in mimicking the implantation process, namely an acute Th1 reaction to facilitate blastocyst adhesion followed by a Th2 modulation to achieve maternal-fetal immune tolerance and controlled blastocyst invasion (Mor et al., 2011). The first landmark study in the field was published by Yoshioka et al. (2006), showing that intrauterine administration of HCG-treated PBMCs could significantly improve reproductive outcomes in women with RIF. Since then a number of studies have been performed so far, with a moderate degree of heterogeneity. The published studies (summarized in Table 3), differ in terms of (a) population characteristics (infertile vs. exclusively RIF patients), (b) the transferred embryos (cleavage embryos vs. blastocysts, fresh vs. frozen embryos), c) the PBMC activation protocol (no-activation, activation by HCG, activation by corticotropin-releasing hormone-CRH). A critical approach in the published literature reveals the fact that although the randomized controlled trials (mainly focusing at RIF) performed (Madkour et al., 2016; Yu et al., 2016; Nobijari et al., 2019; Pourmoghadam et al., 2020b) are comparable to non-randomized trials (Yoshioka et al., 2006; Okitsu et al., 2011; Makrigiannakis et al., 2015; Li et al., 2017b; Makrigiannakis et al., 2019) in terms of quantity, the pooled number of participants is greater for the non-randomized trials. This may question the level of the overall evidence produced.

**TABLE 3:** Salient features of the included studies on PBMC as an intervention in improving endometrial receptivity.

Year	PMID	Publication type	Participants	PBMC activation	RIF	Outcome
2020	32781360	RCT	100	HCG	Yes	Significantly favorable in RIF
2020	31893538	Meta-analysis	1215	HCG	YES	Significantly favorable in RIF
2020	31791175	Meta-analysis	1173	HCG or non-activated	Not exclusively	Significantly favorable in RIF (Sub group analysis)
2019	31322496	RCT	250	CRH	Not exclusively	Significantly favorable in RIF (Sub group analysis)
2019	30739317	Non-randomized	26	CRH	YES	Significantly favorable in RIF
2019	30684765	Meta-analysis	886	HCG or non-activated	Not exclusively	Significantly favorable
2017	27915038	Non-randomized	633	HCG	Not exclusively	Significantly favorable in RIF with cleavage embryos (Sub group analysis)
2016	27521928	RCT	198	HCG	YES	Significantly favorable in RIF
2015	25652716	Non-randomized	45	CRH	YES	Significantly favorable in RIF
2011	22035703	Non-randomized	253	Non-activated	Not exclusively	Significantly favorable in RIF (Sub group analysis)
2006	17021188	Non-randomized	35	HCG	YES	Significantly favorable in RIF

Recently, an array of meta-analyses has emerged, three referring to infertile populations in general (Maleki-Hajiagha et al., 2019; Yakin et al., 2019; Yang et al., 2020) and one referring to patients with RIF (Pourmoghadam et al., 2020a). Of the meta-analysis, evaluating the method as an intervention for infertility in general, the one that supports a significant benefit to the general infertile population (Maleki-Hajiagha et al., 2019) involves fewer participants than the two that draw a non-significant result (Yakin et al., 2019; Yang et al., 2020). Interestingly, subgroup analysis has revealed a significant improvement in reproductive outcomes in women with RIF (Yakin et al., 2019; Yang et al., 2020). The only meta-analysis focused on RIF, having included 1,215 participants, has concluded that intra-uterine administration of PBMC significantly improves the reproductive result in women with RIF (Pourmoghadam et al., 2020a). The results of the meta-analyses, combined, regarding the significantly positive results on RIF, imply a future role for this procedure in treating women with RIF. However, these findings should be treated with caution rather than enthusiasm. The level of evidence is rather weak and thus properly powered randomized trials are needed to enhance the evidence base to an acceptable level.

### Intrauterine Administration of Platelet-Rich Plasma

Platelet-rich-plasma (PRP) is a platelet-rich whole blood extract, having removed red and white blood cells. It is considered an inexpensive means of delivering high concentrations of growth factors since activated platelets release, via their α-granules, high concentrations of VEGF, TGFβ and PDGF (Lang et al., 2017; Baba et al., 2019). As a result PRP is considered effective as a regeneration and anti-inflammatory agent (Vitagliano et al., 2019; Arora and Arora, 2020). Local administration of PRP has been used in several medical fields like orthopedics, otolaryngology and ophthalmology. Five years ago, PRP was successfully applied for the first time as an intervention for improving refractory endometrium of women to receive IVF (Chang et al., 2015). Since then, several case series have been published with promising results. So far, three randomized controlled trials (Eftekhar et al., 2018; Nazari et al., 2020; Zamaniyan et al., 2020) along with two non-randomized controlled trials (Chang et al., 2019; Coksuer et al., 2019) have been published showing significant improvement of the reproductive outcomes. The single meta-analysis in the literature has included seven studies (625 participants) of which 3 were randomized controlled trials and four were cohort studies (Maleki-Hajiagha et al., 2020) (Table 4). One of these four studies compared PRP administration with G-CSF administration while the rest used untreated controls (Maleki-Hajiagha et al., 2020). Of the three RCTs included, one was available as abstract. It was shown that all reproductive outcomes were significantly improved in PRP-treated cases (Maleki-Hajiagha et al., 2020).

**TABLE 4:** Salient features of the included studies on PRP as an intervention in improving endometrial receptivity.

Year	PMID	Publication type	Participants	RIF	Outcome
2020	32363968	RCT	98	Yes	Significantly favorable
2020	30714427	RCT	97	Yes	Significantly favorable
2020	32006776	Meta-analysis	625	No	Significantly favorable
2019	30966843	Non-randomized	34	Yes	Significantly favorable
2019	30653117	Non-randomized	64	No	Significantly favorable
2018	30545532	RCT	83	No	Significantly favorable

The data upon women with RIF are also rather weak. Two randomized controlled trials and one non-randomized controlled trial address the PRP treatment in women with RIF reporting significant improvement in IVF efficacy (Coksuer et al., 2019; Nazari et al., 2020; Zamaniyan et al., 2020), however the patients recruited in total do not allow extraction of definite conclusions. Thus, until evidence of properly designed randomized trials emerge, PRP should be offered within the frame of a trial.

### Granulocyte Colony Stimulating Factor (G-CSF)

The role of G-CSF in reproductive physiology has been mainly studied the last two decades. G-CSF is produced by the granulosa cells during ovulation (Robert et al., 2019). It can be identified in the uterus mainly on the uterine NK cells, which play a major role during implantation both by enhancing receptivity and enabling endometrial synchronization (Sharma and Das, 2014; Robert et al., 2019). More importantly, G-CSF has been shown to regulate Th2 immunity, contributing to maternal-fetal immuno-tolerance (Moldenhauer et al., 2010).

The positive effects exerted by G-CSF on endometrial receptivity and implantation, supported the idea of using G-CSF as a local or systematic immune-modulator during IVF, and thus several observational studies and thereafter randomized and non-randomized clinical trials have emerged. Of note is the heterogeneity of the studies. Different populations selected (infertile, RIF, cases with thin endometrium), different ways of administration (intrauterine, subcutaneously), different concentrations offered and different study endpoints made it difficult to extract a solid conclusion. So far 10 randomized controlled trials have been published (Barad et al., 2014; Aleyasin et al., 2016; Davari-Tanha et al., 2016; Eftekhar et al.,2016a,b; Sarvi et al., 2017; Arefi et al., 2018; Bakirarar et al., 2018; Huang et al., 2020a; Kalem et al., 2020), with conflicting results, followed by meta-analyses in a timely manner (presented in Table 5). The six meta-analyses published present rather supportive results (Zhao et al., 2016; Kamath et al., 2017; Li et al., 2017a; Hou et al., 2018; Jiang et al., 2020; Kamath et al., 2020). However, most of the meta-analyses have included both randomized controlled trials and non-randomized trials, weakening the level of evidence. The most powered meta-analysis at the moment is a Cochrane review including 15 randomized control trials with 1,253 participants (Kamath et al., 2020). The authors have shown a weak positive impact of G-CSF in reproductive outcomes, advising caution due to low quality data and increased uncertainty. As far as RIF cases are concerned, it was found that there could be a benefit by G-CSF administration (Kamath et al., 2020).

**TABLE 5:** Salient features of the included studies on G-CSF as an intervention in improving endometrial receptivity.

Year	PMID	Publication type	Participants	RIF	Outcome
2020	32862740	RCT	163		Significantly favorable
2020	32663652	Meta-analysis	1164	Yes	Significantly favorable in RIF
2020	32198409	RCT	157	Yes	Non-significant
2020	31978254	Meta-analysis	1253	Not exclusively	Significantly favorable in RIF (subgroup analysis)
2019	31091064	Non-randomized	66	Yes	RIF patients did not differ from patients with their 1st IVF cycle
2019	30568355	RCT	150	Not exclusively	Non-significant
2018	30220024	Meta-analysis	1016	Not exclusively	Significantly favorable, Significantly favorable in RIF (subgroup analysis)
2018	30027145	RCT	50	Not exclusively	Non-significant
2017	28632452	Meta-analysis		Not exclusively	Significantly favorable, Significantly favorable in RIF (subgroup analysis)
2017	28458165	Meta-analysis	255	Not exclusively	Significantly favorable Significantly favorable in RIF (subgroup analysis)
2017	27874292	Non-randomized	62	Not exclusively	Non-significant
2017	28791050	RCT	28	Not exclusively	Significantly improved implantation rate
2016	27659067	Meta-analysis	1101	Not exclusively	Significantly favorable (sc) Non-significant (intrauterine)
2016	28066833	RCT	100	Yes	Non-significant pregnancy rate
2016	27981253	RCT	90	Yes	Significantly favorable in RIF
2016	27326420	RCT	100		Non-significant
2016	26980809	RCT	112	Yes	Significantly favorable in RIF
2014	24424357	RCT	141	Not exclusively	Non-significant
2014	25469123	Non-randomized	68	Not exclusively	Non-significant
2014	23885097	Non-randomized	59	Not exclusively	Non-significant

To the same direction most of the meta-analyses have showed a positive impact of G-CSF administration in case of RIF. Due, though, to the poor quality studies included, an increased level of uncertainty is generally noted. This uncertainty is in line with a recent randomized controlled trial showing that in case of RIF patients with normal endometrial thickness, G-CSF does not alter reproductive outcome (Kalem et al., 2020). It is thus evident that G-CSF is not to be applied as infertility intervention in the general population. Even in case of RIF the evidence does not allow G-CSF incorporation to the everyday practice. Properly powered studies are needed to clarify to which group of infertile patients would G-CSF offer some benefit.

### Growth Hormone (GH)

The role of growth hormone in endometrial receptivity is still under investigation. It has been shown that GH receptors are expressed by endometrial epithelium, selectively during the mid-luteal phase (possibly during the window of implantation) and thereafter during decidualization (Sbracia et al., 2004). This expression pattern is similar to other molecules linked to endometrial receptivity. Additionally, it has been recently reported that GH may act, directly or indirectly, as an inducer for VEGF and integrin B3 expression, both involved in endometrial receptivity (Cui et al., 2019). As a result, GH has been demonstrated to be a mediator toward endometrial thickening, being rather appealing as a research intervention for women with thin endometrium (Lan et al., 2019). The evidence supporting GH for treating infertile women stems mainly from studies focusing on poor ovarian responders, mainly due to the parallel action that GH exerts on the ovary (Altmäe and Aghajanova, 2019; Huang et al., 2020b). So far, only one randomized trial has been published studying the GH-impact on women with RIF (Altmäe et al., 2018). The authors report that GH-treated RIF patients presented with significantly thicker endometrium and achieved significantly better reproductive outcomes compared to untreated RIF patients or women receiving their first IVF cycle (Altmäe et al., 2018). Although GH treatment seems promising, the lack of evidence does not allow its use as a standard of care.

### Atosiban

Atosiban is a receptor inhibitor of oxytocin and V1a vasopressin. Based on the observation that embryo-transfer may trigger uterine contractions, which could be detrimental in embryonic apposition, the first case report of a successful pregnancy after atosiban was published in 2007 (Pierzynski et al., 2007). Since then four randomized controlled trials (Table 6) have been published (Moraloglu et al., 2010; Ng et al., 2014; He et al., 2016; Yuan et al., 2019), with the most powered reporting a non-significant effect on reproductive outcomes on the general population (Ng et al., 2014). A recent meta-analysis combining six studies (1,754 participants) shows a rather weak improvement in clinical pregnancy rates but no effect on live birth rates in the general population (Huang et al., 2017b). Of note is the fact that this meta-analysis included both randomized and non-randomized trials, thus weakening the level of evidence of the reported findings. Only two non-randomized studies on RIF patients report a significant benefit after atosiban treatment (Chou et al., 2011; Lan et al., 2012). Interestingly, both the meta-analysis (Huang et al., 2017b) and a recent prospective study on women with at least one IVF effort (Wu et al., 2020), after performing subgroup analysis, report significant improvement on the reproductive outcomes in case of RIF patients. Taking into consideration the fact that subgroup analysis may include a risk of statistical bias, this observation implies a potential role for atosiban when treating women with RIF. Properly powered randomized controlled trials are needed to delineate atosiban efficacy.

**TABLE 6:** Salient features of the included studies on atosiban as an intervention in improving endometrial receptivity.

Year	PMID	Publication type	Participants	RIF	Outcome
2020	32046434	Non-randomized	403	Not exclusively	Significantly favorable in RIF (subgroup analysis)
2019	30791824	RCT	203	Not exclusively	Significantly favorable in case of difficult ET
2017	28422984	Meta-analysis	1754	Not exclusively	Significantly favorable in RIF (subgroup analysis)
2016	27143518	RCT	240	Not exclusively	Significantly favorable
2014	25336707	RCT	800	Not exclusively	Non-significant
2012	22818095	Non-randomized	71	Yes	Significantly favorable in RIF
2011	21791296	Non-randomized	150	Yes	Significantly favorable in RIF
2010	20638340	RCT	180	Not exclusively	Significantly favorable

### Antibiotics for Chronic Endometritis

The field of chronic endometritis (CE) is emerging, especially in unexplained RIF, since CE presents a subtle course that can be easily missed. Few reports have been published so far upon antibiotic treatment in case of CE diagnosed in women with unexplained RIF (Johnston-MacAnanny et al., 2010; Yang et al., 2014; Cicinelli et al., 2015; Tersoglio et al., 2015; Bouet et al., 2016; Kitaya et al., 2017; Zhang et al., 2019). Analyzing this therapeutic approach, it can be easily seen that there is substantial heterogeneity in terms of antibiotic treatment: antibiotics are offered according to microbial cultures or empirically, including penicillins, cephalosporins, kinolones, metronidazole, clindamycin, tetracyclines (mainly doxycycline), and aminoglycodides (gentamycine). The course of treatment also varies. Interestingly, apart from the standard *per os* treatment, the intrauterine approach has also been presented (Zhang et al., 2019). All the studies published so far are non-randomized thus producing low level of evidence. Two recent meta-analyses have summarized the existing results, supporting antibiotic treatment as an approach to improve reproductive outcomes in women with RIF (Vitagliano et al., 2018b; Huang et al., 2020b). Additionally, it is highlighted that a stringent approach in setting the diagnosis may reveal lower CE incidences and reproductive outcomes compared to approaches with broader diagnostic criteria (Huang et al., 2020b). Long-term treatments seem more beneficial compared to short-term antibiotic courses (Huang et al., 2020b).

### Efforts to Intervene in Case of Endometrial Dysbiosis

The concept of endometrial dysbiosis is emerging during the last 5 years. Several approaches have been proposed as potentially effective in restoring normal endometrial microbiome. However it must be taken under consideration the fact that to date there is no core endometrial microbiome, therefore restoring to normal could be a matter of question (Molina et al., 2020). Microbiome restoration is initially approached by the administration of antibiotics, considering that most of the time dysbiotic microbiota may include pathogens well-controlled by standard anti-microbial agents (Kyono et al., 2019). Several routes of administration have been proposed so far including oral, vaginal and intrauterine (Molina et al., 2020). Additionally, the administration of pro- and pre-biotics has been tested as auxiliary means of maintaining or amplifying the eubiotic bacteria. Of note is the mode of action of pro-biotics—bacteria involved in normal microbiome, administered in the context to colonize in an antagonizing fashion the dysbiotic microbial counterparts (Chenoll et al., 2019). On the contrary, pre-biotics are molecules uptaken by normal microbial populations, facilitating their survival. Pre- and pro-biotics are usually co-administered with antibiotics. A recent study investigated different routes of antibiotic administration (metronidazole) combined with prebiotic (lactoferrin) and probiotic administration, concluded that the combined vaginal and oral metronidazole administration along with a vaginal probiotic treatment could restore normal endometrial microbiome in women with RIF (Kadogami et al., 2020). New approaches have been proposed as promising, lacking evidence at the moment. Vaginal microbiome transplants have been considered as a possibility. A recent study showed the restoration of the vaginal microbiome in case of refractory vaginosis (Lev-Sagie et al., 2019), a fact that could imply further colonization of the endometrial cavity by the ascending route. Taking all the above into consideration, it is clear that there is no evidence at the moment to support both endometrial microbiome assessment and thereafter interventions toward restoration to normal. Such efforts have to be strictly performed in the frame of a research protocol.

## Conclusion

All the novel interventions, aiming to treat unexplained RIF, lack the evidence required in order to be incorporated to standard of care. Properly designed randomized trials are therefore needed to clarify which could be beneficial in RIF treatment. RIF patients should be properly informed regarding potential benefits and risks.

## Author Contributions

AM had the idea for this review. AM, FM, and TV participated in literature search, drafted, and critically revised the manuscript, approved the final version of the manuscript. All authors contributed to the article and approved the submitted version.

## Conflict of Interest

The authors declare that the research was conducted in the absence of any commercial or financial relationships that could be construed as a potential conflict of interest.
